# Kv1.1 deficiency alters repetitive and social behaviors in mice and rescues autistic‐like behaviors due to *Scn2a* haploinsufficiency

**DOI:** 10.1002/brb3.2041

**Published:** 2021-01-23

**Authors:** Jagadeeswaran Indumathy, April Pruitt, Nicole M. Gautier, Kaitlin Crane, Edward Glasscock

**Affiliations:** ^1^ Department of Cellular Biology and Anatomy Louisiana State University Health Sciences Center Shreveport LA USA; ^2^ Present address: Department of Biological Sciences Southern Methodist University Dallas TX USA

**Keywords:** autism spectrum disorder, channelopathies, comorbidity, epilepsy, ion channels

## Abstract

**Background:**

Autism spectrum disorder (ASD) and epilepsy are highly comorbid, suggesting potential overlap in genetic etiology, pathophysiology, and neurodevelopmental abnormalities; however, the nature of this relationship remains unclear. This work investigated how two ion channel mutations, one associated with autism (*Scn2a*‐null) and one with epilepsy (*Kcna1*‐null), interact to modify genotype–phenotype relationships in the context of autism. Previous studies have shown that *Scn2a* ^+/–^ ameliorates epilepsy in *Kcna1* ^–/–^ mice, improving survival, seizure characteristics, and brain–heart dynamics. Here, we tested the converse, whether *Kcna1* deletion modifies ASD‐like repetitive and social behaviors in *Scn2a^+/–^* mice.

**Methods:**

Mice were bred with various combinations of *Kcna1* and *Scn2a* knockout alleles. Animals were assessed for repetitive behaviors using marble burying, grooming, and nestlet shredding tests and for social behaviors using sociability and social novelty preference tests.

**Results:**

Behavioral testing revealed drastic reductions in all repetitive behaviors in epileptic *Kcna1* ^–/–^ mice, but relatively normal social interactions. In contrast, mice with partial *Kcna1* deletion *(Kcna1* ^+/–^) exhibited increased self‐grooming and decreased sociability suggestive of ASD‐like features similar to those observed in *Scn2a* ^+/–^ mice. In double‐mutant *Scn2a* ^+/–^; *Kcna1* ^+/–^ mice, the two mutations interacted to partially normalize ASD‐like behaviors associated with each mutation independently.

**Conclusions:**

Taken together, these findings suggest that Kv1.1 subunits are important in pathways and neural networks underlying ASD and that *Kcna1* may be a therapeutic target for treatment of *Scn2a*‐associated ASD.

## Graphical abstract

This work investigates how two ion channel mutations, one associated with autism (*Scn2a*‐null) and one with epilepsy (*Kcna1*‐null), interact to modify genotype–phenotype relationships in the context of autism. Behavioral testing revealed altered repetitive and social behaviors in mice dependent on whether the *Kcna1* gene was partially or completely knocked out. In double‐mutant *Scn2a* ^+/–^; *Kcna1* ^+/–^ mice, the two mutations interacted to partially rescue ASD‐like behaviors associated with each mutation independently, suggesting that Kv1.1 subunits are important in pathways and neural networks underlying ASD and that *Kcna1* may be a therapeutic target for treatment of *Scn2a*‐associated ASD.

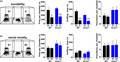

## 1 INTRODUCTION

Autism spectrum disorder (ASD) and epilepsy are common neurological diseases that often co‐occur in patients for reasons that are not well understood. The two main hallmarks of ASD are impaired social interaction and communication and the presence of restricted repetitive behavior, whereas epilepsy is characterized by the presence of spontaneous recurrent seizures (Stafstrom & Carmant, 2015; de la Torre‐Ubieta et al., 2016). About 6%–27% of people diagnosed with ASD also have epilepsy, and there is a sevenfold increased risk of epilepsy in ASD patients (Jeste & Tuchman, 2015; Thomas et al., 2017). Similarly, about 37% of children <5 years of age with epilepsy test positive for autism, and there is a 6‐ to 8‐fold increased risk of ASD in epilepsy patients overall (Fisher et al., 2012; Strasser et al., 2018). One explanation for the high comorbidity rate of ASD and epilepsy is that the two disorders have a common underlying neuropathophysiology, which could be caused by a shared genetic basis since many genes associated with epilepsy are also linked to autism (Jeste & Tuchman, 2015; Srivastava & Sahin, 2017).

In addition to rare monogenic causes, both ASD and epilepsy have significant genetic etiologies involving complex interactions between multiple genetic variants which can be inherited or arise de novo (Klassen et al., 2011; de la Torre‐Ubieta et al., 2016). Although these mutations are typically thought to add together in a deleterious fashion causing disease when some risk threshold is crossed, the opposite can also be true, that mutations can exert protective effects that mask disease (Devlin & Scherer, 2012; Noebels, 2015; de la Torre‐Ubieta et al., 2016). Indeed, in both ASD and epilepsy, beneficial suppressor mutations have been identified that are capable of ameliorating the harmful effects of known disease‐causing variants (Glasscock et al., 2007; Leblond et al., 2012). Protective genetic modifiers represent valuable opportunities for improving diagnostic risk assessment, identifying new therapeutic targets, and revealing underlying disease mechanisms. Complex interplay between mutations associated with epilepsy and autism (i.e., epistatic effects) could partially explain the high degree of comorbidity for the two conditions, as well as the absence of disease when high‐risk variants are present.

One gene that has come to light as an important molecular cause of both epilepsy and autism is *Scn2a*, which encodes neuronal Nav1.2 voltage‐gated sodium channel α‐subunits that regulate action potential initiation and propagation in axons and action potential backpropagation in somatodendritic compartments (Hu et al., 2009; Spratt et al., 2019). Nav1.2 is widely expressed throughout the central nervous system with subcellular localization in axons and somatodendritic regions, including unmyelinated axons and axon initial segments (AIS) of excitatory neurons (and some interneurons) (Li et al., 2014; Sanders et al., 2018). Loss‐of‐function mutations in *Scn2a* are strongly associated with ASD and intellectual disability in patients, whereas gain‐of‐function mutations cause encephalopathy with early‐onset infantile epilepsy (Sanders et al., 2012, 2018). In some individuals, *Scn2a* mutations can lead to both epilepsy and autistic features (Kamiya et al., 2004; Wolff et al., 2017). Several recent studies have shown that heterozygous *Scn2a* knockout mice (*Scn2a* ^+/–^) faithfully model features of autism without epilepsy, including impaired social behavior and synaptic dysfunction (Léna & Mantegazza, 2019; Spratt et al., 2019; Tatsukawa et al., 2019).

In addition to causing autistic‐like phenotypes, heterozygosity for the *Scn2a* knockout allele (i.e., *Scn2a* ^+/–^) also acts as a protective modifier of epilepsy and sudden death in mice carrying a deletion of the *Kcna1* gene (i.e., *Kcna1* ^–/–^), which encodes Kv1.1 voltage‐gated potassium channel α‐subunits that regulate axonal excitability (Mishra et al., 2017). In *Kcna1* ^–/–^ mice, the *Scn2a* ^+/–^ mutation increases survival, decreases seizure durations, and improves brain–heart dynamics, likely as a result of the channels’ complementary functions and expression patterns (Mishra et al., 2017). Given the beneficial epistatic interactions between these two ion channel mutations in the context of epilepsy, in this work a battery of behavioral tests was employed to investigate whether the *Scn2a* and *Kcna1* mutations interact to modify genotype–phenotype relationships in the context of autism. First, we examined whether *Kcna1* ^–/–^ mice exhibit autistic‐like tendencies in measures of repetitive and social behaviors. Second, we generated double‐mutant *Scn2a* ^+/–^ mice with various combinations of *Kcna1*‐null alleles to test the hypothesis that *Kcna1* deletion can act as a protective modifier of *Scn2a*‐related autistic‐like phenotypes. The results of this study provide a window into the types of complex genetic interactions underlying autism–epilepsy comorbidity, while also identifying *Kcna1* as a modifier of ASD susceptibility and Kv1.1 subunits or related networks and pathways as potential therapeutic targets in autism due to *Scn2a* mutations.

## 2 MATERIALS AND METHODS

### 2.1 Animals and genotyping

The *Kcna1* knockout (KO) allele was generated by targeted deletion of the entire open reading frame of the *Kcna1* gene (chromosome 6), as described previously (Smart et al., 1998). *Kcna1* mice are maintained on a Black Swiss (Tac:N:NIHS‐BC) genetic background. The initial behavioral analyses of *Kcna1* ^−/−^ knockout mice and controls described in the results were performed on animals from the Tac:N:NIHS‐BC genetic background. The *Scn2a* KO allele was created by targeted deletion of the first half of exon 1 of the *Scn2a* gene (chromosome 2), as described previously (Planells‐Cases et al., 2000). *Scn2a* mice are maintained on a C57BL/6J background. Double‐mutant mice carrying various combinations of *Scn2a* and *Kcna1* null alleles were generated by crossing F1 double heterozygotes (*Scn2a* ^+/–^; *Kcna1* ^+/–^) in a mixed Black Swiss (Tac:N:NIHS‐BC) x C57BL/6J genetic background. The F1 double heterozygotes were obtained by crossing heterozygous *Scn2a*‐null (*Scn2a* ^+/–^; C57BL/6J background) mice and heterozygous *Kcna1*‐null (*Kcna1* ^+/–^; Tac:N:NIHS‐BC background) mice. It should be noted that a limitation of this crossing strategy is that it generates double‐mutant mice for experiments that have mixed percentages of the two genetic backgrounds. Although these percentages should be similar between animals, they are not identical and could influence the outcome of the behavioral tests. Mice were group‐housed at ~22°C, fed ad libitum, and submitted to a 12‐hr light/dark cycle. All procedures were performed in accordance with the guidelines of the National Institutes of Health (NIH), as approved by the Institutional Animal Care and Use Committee of the Louisiana State University Health Sciences Center‐Shreveport.

For genotyping, genomic DNA was isolated by enzymatic digestion of tail clips using Direct PCR Lysis Reagent (Viagen Biotech). Genotypes were determined by performing PCR amplification of genomic DNA using allele‐specific primers. For *Kcna1*, the following primer sequences were used to yield amplicons of ~337 bp for the WT allele and ~475 bp for the KO allele: a KO‐specific primer (5’‐CCTTCTATCGCCTTCTTGACG‐3’), a WT‐specific primer (5’‐GCCTCTGACAGTGACCTCAGC‐3’), and a common primer (5’‐GCTTCAGGTTCGCCACTCCCC‐3’). For *Scn2a*, the following primer sequences were used to yield amplicons of ~450 bp for the WT allele and ~1,300 bp for the KO allele: a forward sense primer (5’‐TGCGAGGAGCTAAACAGTGATTAAAG‐3’) and a reverse antisense primer (5’‐ GGCTCCATTCCCTTATCAG ACCTACCC‐3’).

In behavioral experiments, age‐matched juvenile mice of both sexes were tested at 28–34 days old. The total number of animals tested was between 6 and 12 per genotype per experiment, as specified. To obtain greater statistical power, mice of both sexes were grouped for analyses. For tests of repetitive behavior, 2–10 males and 2–7 females per genotype were used. For tests of social interactions, 3–9 males and 1–5 females per genotype were used. The exact numbers of mice of each sex per genotype are indicated in the figure legends. All experiments were conducted during the daytime and analyzed by individuals blinded to genotype. Animals displaying overt behavioral seizure activity immediately before or during behavioral assessment were excluded from analysis; however, only *Kcna1* ^−/−^ and *Scn2a* ^+/–^; *Kcna1* ^–/–^ mice exhibit seizures. Animals were exposed to each test one time over 3 days in the following order: marble burying (day 1); self‐grooming (day 1, 25 min after marble burying test); sociability and social novelty (day 2); and nestlet shredding (day 3).

### 2.2 Marble burying

Mice were individually placed in a standard shoebox rat cage (~143‐in^2^ floor space; Allentown, Inc.) containing a 4‐cm layer of fresh bedding (Sani‐Chips; Lab Supply) with 20 marbles arranged in 5 equally spaced rows of 4 marbles each (i.e., a 4 × 5 grid pattern), as previously described (Angoa‐Pérez et al., 2013). Marbles were scored as buried if they were at least two‐thirds covered at the end of a 30‐min test session. The number of buried marbles was scored by two individuals independently, and their results averaged to give a value for each animal.

### 2.3 Nestlet shredding

Mice were individually placed in a standard shoebox mouse cage (~75‐in^2^ floor space; Allentown, Inc.) with an intact preweighed cotton nestlet (2‐in square; Lab Supply) and left overnight for 16 hr. At the end of the session, the remaining intact (nonshredded) portion of the cotton nestlet was removed from the cage, allowed to dry overnight, and weighed. The percentage of the nestlet shredded was then calculated using the measurements of the intact nestlet mass before and after introduction of the mouse, as previously described (Angoa‐Pérez et al., 2013).

### 2.4 Self‐grooming

Mice were individually placed in a standard shoebox mouse cage with ~1 cm of fresh bedding (Sani‐Chips; Lab Supply) and allowed to habituate for 5 min. Then, the mice were observed for 15 min to quantify grooming behaviors, as previously described (Silverman et al., 2010). Grooming behavior was defined as stroking or scratching of face, head, or body with two forelimbs, or licking body parts. Total grooming time was scored by two trained observers independently using a stopwatch, and the average for each animal was calculated.

### 2.5 Sociability and social novelty

Mice were tested for social interactions using a rectangular plexiglass three‐chamber box (66‐cm length × 45‐cm width × 22‐cm height), composed of a middle chamber separated by dividing walls with retractable doorways from two flanking and equal‐sized side chambers (20‐cm length × 45‐cm width), as previously described (Crawley, 2007; Moy et al., 2004). First, the test mouse was placed in the middle chamber for 5 min to habituate. Then, a stranger mouse (S1) was placed in one of the side chambers under an inverted wire pencil cup (10‐cm diameter × 10‐cm height). In the other, side chamber was placed an empty inverted wire pencil cup (E) as a nonsocial object. The doorways were then raised, and the test mouse allowed to explore the side chambers for 10 min. The inverted wire pencil cups were composed of vertical wires that were spaced widely enough (~7 mm) to allow the test mouse to see and sniff the stranger mouse while preventing aggressive or sexual interactions. Plastic cups with weights in them were placed on top of the inverted pencil cups to prevent them from moving during the test. Sociability was quantified as the preference of the test mouse for the stranger mouse (S1) versus the inanimate object alone (E). For testing social novelty, immediately at the conclusion of the sociability test, a second stranger mouse (S2) was placed under the empty inverted wire pencil cup. The test mouse was then allowed access to the side chambers for another 10 min. Social novelty was measured as the preference of the test mouse for the new stranger mouse (S2) compared to the previous stranger mouse (S1).

For both tests, the following variables were measured during the 10‐min trial periods to quantify chamber preference and social interaction: the total time spent in each chamber; the total time the test mouse spent sniffing the stranger mouse or the empty wire cup; and the number of entries into each chamber. The time spent in each chamber provides a general gauge of interest, while sniffing time gives a more specific measure of social investigation. The number of chamber entries between compartments provides a general measure of locomotion and activity. Times and counts were measured manually by offline video observation performed by two individuals blinded to genotype whose results were then averaged to give a value for each animal. Two mice (one *Scn2a* ^+/–^; *Kcna1* ^–/–^ mouse and one WT mouse from the mixed background) that failed to explore each of the two side chambers for a minimum of 90 s (15% of the total test time) were excluded from analysis.

Stranger mice were age‐ and sex‐matched to the test mice. In addition, the stranger mice (which were selected to always have a *Kcna1* ^+/–^ genotype) were from different litters than the test mice, and they were never housed together with the test mice or with the other stranger mice used in the same experiment. The location of stranger mice was alternated between the left and right chambers for each experiment for a given genotype to eliminate potential side bias. New clean wire pencil cups were used for each experiment, and the surfaces of the three‐chamber box were wiped clean with Clidox in between animals.

### 2.6 Statistical analysis

Statistical analysis was performed with Prism 8 for Windows (GraphPad Software Inc, La Jolla, CA), and the data are expressed as means ± standard error of the mean (*SEM*). The normality of the data was checked using the D’Agostino–Pearson test. For comparison of repetitive behavior and social interaction, unpaired two‐tailed Student's *t* tests (involving only two groups) and one‐way ANOVA with Tukey's multiple comparison *post hoc* tests (involving three or more groups) were performed, as appropriate. *p* Values < .05 were considered statistically significant.

## 3 RESULTS

### 3.1 *Kcna1* ^–/–^ mice exhibit altered repetitive behavior

**FIGURE 1:**
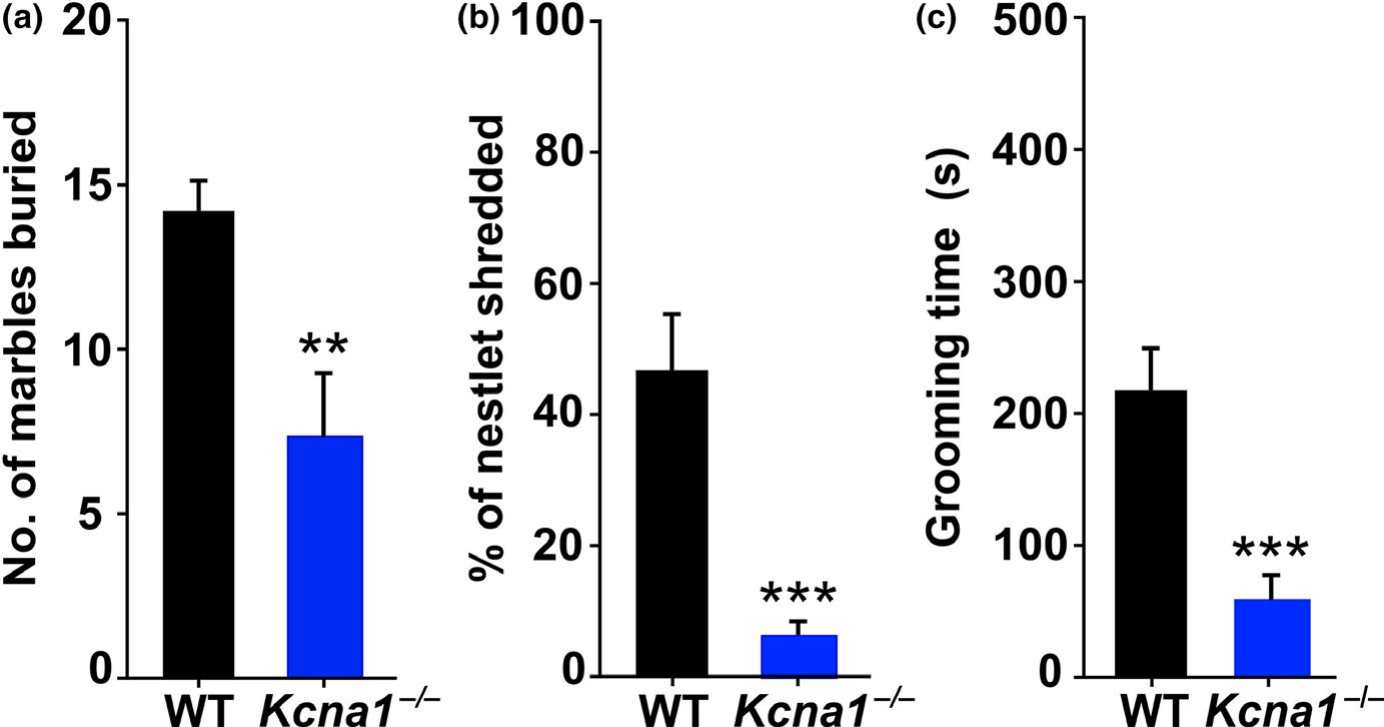
*Kcna1* ^–/–^ mice exhibit drastic reductions in repetitive behaviors. Repetitive behaviors of *Kcna1* ^–/–^ mice (*n* = 12:5 males, 7 females) and wild‐type (WT) control animals (*n* = 13:6 males, 7 females) were evaluated in tests of marble burying (a), nestlet shredding (b), and self‐grooming (c). **, *p* < .01; ***, *p ≤ *.001 (unpaired two‐tailed Student's *t* test)

To determine whether epileptic *Kcna1*‐null mice (*Kcna1* ^–/–^; Tac:N:NIHS‐BC genetic background) exhibit abnormal repetitive behaviors, age‐matched juvenile *Kcna1^–/–^* mice (*n* = 12) and WT littermates (*n* = 13) were evaluated in tests commonly used for assessing autistic‐like repetitive behaviors: marble burying, nestlet shredding, and self‐grooming abilities (Angoa‐Pérez et al., 2013; Kim et al., 2016). In tests of marble burying–digging behavior, *Kcna1* ^–/–^ mice buried about 50% fewer marbles (7.3 ± 1.9 marbles) compared to WT controls (14.2 ± 0.9 marbles; two‐tailed Student's *t* test, *t*(23) = 3.28, *p* = .0033; Figure 1a). In addition, nestlet shredding behavior was almost completely absent in *Kcna1* ^–/–^ mice, which only shredded 6.0 ± 2.4% of a cotton nestlet during the overnight testing period compared to 46.8 ± 8.5% in WT mice (two‐tailed Student's *t* test, *t*(23) = 4.44, *p* = .0002; Figure 1b). Finally, when placed in a new cage, *Kcna1* ^–/–^ mice spent about 74% less time self‐grooming (57.3 ± 19.3 s) compared to their WT littermates (217.4 ± 32.1 s; two‐tailed Student's *t* test, *t*(23) = 4.19, *p* = .0003; Figure 1c). Thus, *Kcna1* ^–/–^ mice exhibited a drastic overall reduction in repetitive behavior for all measures tested, suggesting that the absence of Kv1.1 could lead to functional changes that oppose autistic‐like behavioral abnormalities. However, their reduced marble burying and nestlet shredding could also be interpreted as evidence for restricted interests, as observed in other mouse models of ASD and in patients (Bernard et al., 2015; Greco et al., 2013).

### 3.2 *Kcna1* ^–/–^ mice exhibit normal social interactions

**FIGURE 2:**
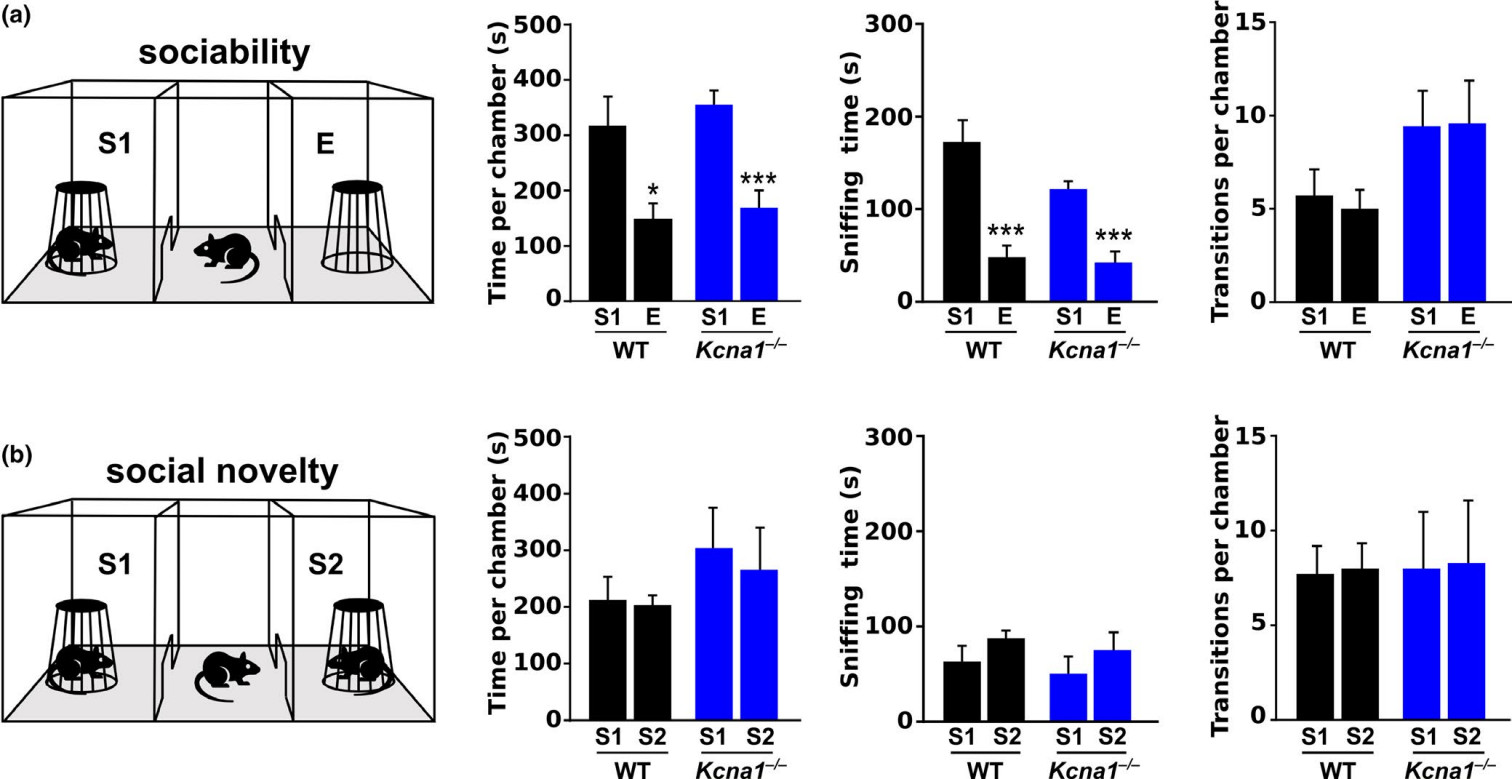
*Kcna1* ^–/–^ mice exhibit normal social interactions. Social interactions were evaluated for WT (*n* = 7:3 male, 4 females) and *Kcna1* ^–/–^ mice (*n* = 7:3 males, 4 females) using three‐chamber tests of sociability (a) and social novelty preference (b). In the sociability test, both genotypes exhibited a preference for the stranger mouse (S1) over the empty cup (E) as evidenced by the significantly greater amount of time spent in the S1 chamber (versus the E chamber) and in sniffing the S1 mouse (versus the empty cup). In the subsequent social novelty test, the preference for the original S1 mouse disappeared with the introduction of a second novel stranger mouse (S2) as both genotypes spent similar amounts of time exploring the side chambers and sniffing both stranger mice. Both genotypes exhibited a similar number of transitions from the middle chamber into the two side chambers suggesting no innate side preference. *, *p* < .05; ***, *p* < .01 (unpaired two‐tailed Student's *t* test)

To determine whether *Kcna1* ^–/–^ mice (Tac:N:NIHS‐BC genetic background) exhibit abnormal social interactions, sociability and an interest in social novelty were assessed using a three‐chamber social approach test. Three‐chamber tests are routinely used for the assessment of ASD‐like social deficits in mice (Kaidanovich‐Beilin et al., 2011; Nadler et al., 2004; Silverman et al., 2012). In the sociability phase of the examination, the preference of the mouse was compared for a novel social object (a stranger mouse in an inverted wire cup; S1) versus a novel nonsocial object (an empty inverted wire cup; E). In tests of sociability, both *Kcna1* ^–/–^ (*n* = 7) and WT mice (*n* = 7) spent significantly more time exploring the chamber containing the stranger mouse than the chamber with the empty cup, indicative of normal social behavior in the animals (two‐tailed Student's *t* test, WT: *t*(12) = 2.83, *p* = .015; *Kcna1* ^–/–^: *t*(12) = 4.65, *p* = .0006; Figure 2a). In addition, both genotypes spent significantly more time sniffing the cup with the mouse in it than the empty cup, further suggesting a preference for social interaction (two‐tailed Student's *t* test, WT: *t*(12) = 4.67, *p* = .0005; *Kcna1* ^–/–^: *t*(12) = 5.40, *p* = .0002; Figure 2a). Finally, *Kcna1* ^–/–^ and WT mice both exhibited similar numbers of entries into each of the side chambers demonstrating similar levels of exploratory activity and a lack of innate side preference. Thus, *Kcna1* ^–/–^ mice exhibited normal sociability.

In the social novelty phase of the experiment, the preference of the test mouse was compared for the original stranger mouse (S1) versus a novel stranger mouse (S2). *Kcna1* ^–/–^ mice (*n* = 7) spent similar amounts of time in each of the two side chambers suggesting a lack of preference for the unfamiliar mouse (two‐tailed Student's *t* test, *t*(12) = 0.37, *p* = .72). However, WT mice (*n* = 7) also unexpectedly exhibited a similar lack of preference (two‐tailed Student's *t* test, *t*(12) = 0.20, *p* = .85; Figure 2b), which limits the interpretation of social novelty features in *Kcna1* ^–/–^ mice. Both genotypes exhibited increased time spent sniffing the new stranger mouse (S2), but these differences did not reach statistical significance (two‐tailed Student's *t* test, WT: *t*(12) = 1.31, *p* = .21; *Kcna1* ^–/–^: *t*(12) = 0.95, *p* = .36; Figure 2b). The number of transitions into the two side chambers was indistinguishable between genotypes suggesting normal exploration. Thus, *Kcna1* ^–/–^ exhibit an interest in social novelty similar to their WT littermates.

### 3.3 ***Scn2a*** ^+^ **^/–^ mice exhibit repetitive and social behavioral abnormalities**

To test whether *Kcna1* gene deletion acts as a genetic modifier of autistic‐like behaviors in *Scn2a* ^+/–^ mice, double‐mutant mice were generated by crossing *Kcna1* ^+/–^ mice (Tac:N:NIHS‐BC genetic background) with *Scn2a* ^+/–^ mice (C57BL/6J genetic background) to yield *Scn2a* ^+/–^ animals with the *Kcna1* gene either completely or partially deleted (i.e., *Scn2a* ^+/–^ *; Kcna1* ^+/–^ or *Scn2a* ^+/–^ *; Kcna1* ^–/–^ on a mixed Tac:N:NIHS‐BC x C57BL/6J genetic background). Mice with various allelic combinations of *Kcna1* and *Scn2a* were then subjected to the same battery of tests for repetitive and social behaviors as performed above.

**FIGURE 3:**
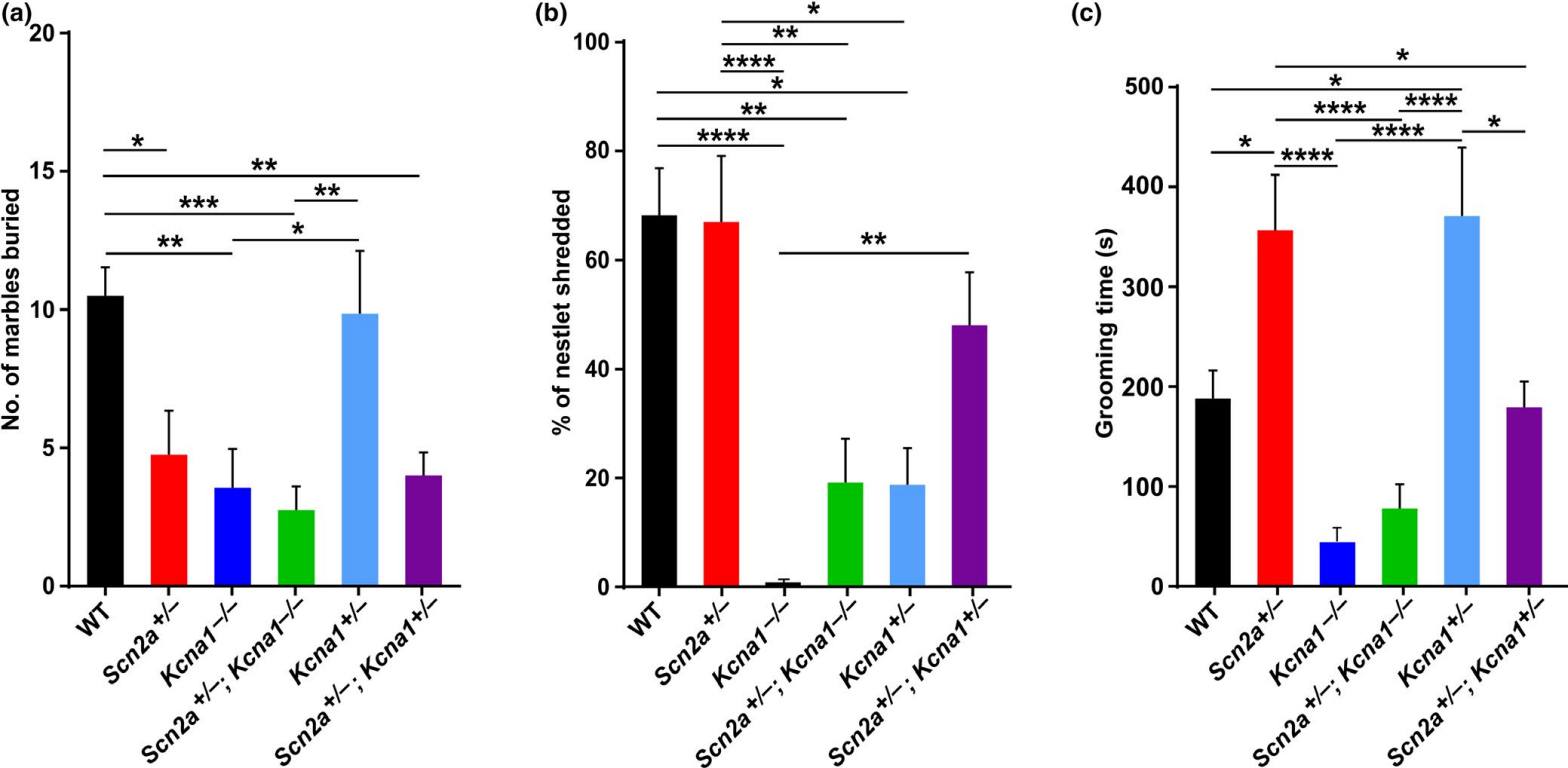
Partial deletion of the *Kcna1* gene rescues abnormal self‐grooming in *Scn2a* ^+/–^ mice. Repetitive behaviors were evaluated in tests of marble burying (a), nestlet shredding (b), and self‐grooming (c). Both *Scn2a* ^+/–^ and *Kcna1* ^+/–^ mice exhibited significantly increased self‐grooming consistent with ASD‐like behavior. However, abnormal self‐grooming behavior was suppressed in double heterozygotes carrying both mutations (i.e., *Scn2a* ^+/–^; *Kcna1* ^+/–^) indicative of reciprocal masking interactions between the two genes. Sample sizes per genotype were the following: for WT, *n* = 12 (9 males, 3 females); for *Scn2a* ^+/–^, *n* = 12 (7 males, 5 females); for *Kcna1* ^–/–^, *n* = 9 (3 males, 6 females); for *Scn2a* ^+/–^; *Kcna1* ^–/–^; *n* = 12 (10 males, 2 females), for *Kcna1* ^+/–^, *n* = 7 (2 males, 5 females); and for *Scn2a* ^+/–^; *Kcna1* ^+/–^, *n* = 12 (6 males, 6 females). *, *p* < .05; **, *p* < .01; ***, *p* < .001; ****, *p* < .0001 (one‐way ANOVA with post hoc Tukey test)

**FIGURE 4:**
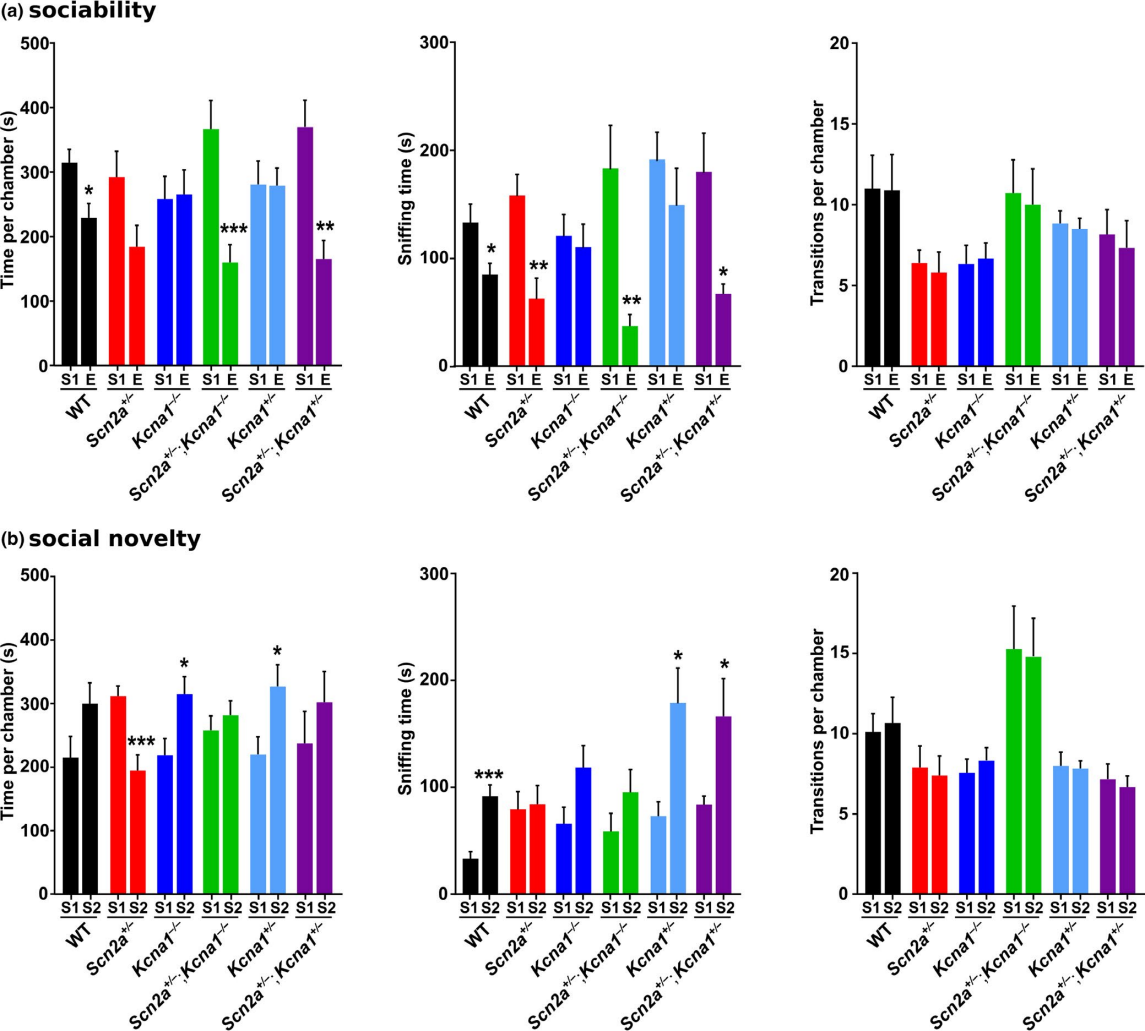
Partial deletion of the *Kcna1* gene rescues deficits in social novelty preference in *Scn2a* ^+/–^ mice. Social interactions were evaluated using three‐chamber tests of sociability (a) and social novelty preference (b). *Scn2a* ^+/–^ mice (*n* = 10) exhibited relatively normal sociability but an abnormal decrease in social novelty preference, as evidenced by spending a significantly greater amount of time exploring the chamber with a familiar stranger mouse (S1) compared to the chamber with a novel stranger mouse (S2). *Kcna1* ^+/–^ mice (*n* = 6) displayed abnormal sociability by exhibiting no significant preference for the stranger mouse (S1) over the empty cup (E) in terms of time spent in each chamber and sniffing time. However, in double heterozygotes carrying both mutations (i.e., *Scn2a* ^+/–^; *Kcna1* ^+/–^; *n* = 6) sociability and social novelty preference were normalized, further suggesting reciprocal masking interactions between the two genes. All genotypes exhibited a similar number of transitions from the middle chamber into the two side chambers suggesting no innate side preference. Sample sizes per genotype were the following: for WT, *n* = 9 (7 males, 2 females); for *Scn2a* ^+/–^, *n* = 10 (5 males, 5 females); for *Kcna1* ^–/–^, *n* = 9 (5 males, 4 females); for *Scn2a* ^+/–^; *Kcna1* ^–/–^; *n* = 11 (9 males, 2 females), for *Kcna1* ^+/–^, *n* = 6 (4 males, 2 females); and for *Scn2a* ^+/–^; *Kcna1* ^+/–^, *n* = 6 (5 males, 1 female). Sample sizes were 6–11 mice per genotype. *, *p* < .05; **, *p* < .01; ***, *p* < .001 (unpaired two‐tailed Student's *t* test)

First, the *Scn2a* ^+/–^ mice were evaluated since previous studies have demonstrated they exhibit an array of autistic‐like behaviors with a penetrance and expressivity that can vary between studies (Léna & Mantegazza, 2019; Spratt et al., 2019; Tatsukawa et al., 2019). In tests of repetitive behavior, *Scn2a* ^+/–^ mice (*n* = 12) displayed significant 55% reductions in marble burying (1‐way ANOVA, *F*(5,58) = 6.70, *p* < .0001; Tukey's post hoc, *q*(58) = 4.75, *p* = .017; Figure 3a) and 53% increases in self‐grooming (1‐way ANOVA, *F*(5,58) = 11.70, *p* < .0001; Tukey's post hoc, *q*(58) = 4.70, *p* = .018; Figure 3c) compared to WT animals (*n* = 12), but their nestlet shredding was similar to WT levels (1‐way ANOVA, *F*(5,58) = 9.17, *p* < .0001; Tukey's post hoc, *q*(58) = 0.14, *p* > .99; Figure 3b). In social interaction tests, *Scn2a* ^+/–^ mice (*n* = 10) exhibited relatively normal sociability, but abnormal social novelty preferences compared to WT (*n* = 9). In the sociability experiments, *Scn2a* ^+/–^ mice showed increased interest in the chamber with the stranger mouse versus the empty cup, but this difference did not reach statistical significance (two‐tailed Student's *t* test, *t*(18) = 2.09, *p* = .052; Figure 4a); however, they did spend significantly more time sniffing the stranger mouse versus the empty cup suggesting normal sociability (two‐tailed Student's *t* test, *t*(18) = 3.41, *p* = .0031; Figure 4a). In the tests of social novelty, *Scn2a* ^+/–^ mice failed to show a preference for the novel stranger mouse over the original familiar stranger mouse, spending significantly more time in the chamber with the original mouse (two‐tailed Student's *t* test, *t*(18) = 3.97, *p* = .0009; Figure 4b). In addition, they sniffed both the new and old stranger mice for similar amounts of time further demonstrating an abnormal lack of interest in the socially novel animal. The number of entries into the side chambers was the same for each social interaction test in *Scn2a* ^+/–^ mice, as well as for all other genotypes detailed below (Figure 4). Thus, the increased repetitive self‐grooming behavior and abnormal interest in social novelty in *Scn2a* ^+/–^ mice are consistent with autistic‐like features, providing further support for their utility as a mouse model of autism. In addition, their significant reductions in marble burying could be a correlate of the restricted interests observed in ASD.

### 3.4 ***Kcna1* deletion ameliorates repetitive and social behavioral deficits in *Scn2a*** ^+^ **^/–^ mice**

In *Scn2a* ^+/–^ *; Kcna1* ^+/–^ mice, heterozygous *Kcna1* gene deletion (i.e., *Kcna1* ^+/–^) normalized the abnormal grooming (1‐way ANOVA, *F*(5,58) = 11.70, *p* < .0001; Tukey's post hoc, *q*(58) = 0.24, *p* > .99; Figure 3c) and social novelty sniffing behavioral deficits (two‐tailed Student's *t* test, *t*(10) = 2.29, *p* = .045; Figure 4b) present in *Scn2a* ^+/–^ animals to WT levels indicative of a potential protective genetic modifier effect. However, marble burying in *Scn2a* ^+/–^ *; Kcna1* ^+/–^ mice remained reduced relative to WT (1‐way ANOVA, *F*(5,58) = 6.70, *p* < .0001; Tukey's post hoc, *q*(58) = 5.37, *p* = .0045; Figure 3a). The ability of *Kcna1* gene deletion to act as a protective modifier of behavioral abnormalities in *Scn2a* ^+/–^ mice was dependent on gene dosage since *Scn2a* ^+/–^ *; Kcna1* ^–/–^ mice showed behavioral phenotypes that were a mix of the *Scn2a* ^+/–^ and *Kcna1* ^–/–^ single mutants (see Table 1 for summary). *Scn2a* ^+/–^ *; Kcna1* ^–/–^ mice still showed marble burying and social novelty deficits compared to WT, like *Scn2a* ^+/–^ mice, as well as reductions in nestlet shredding and grooming similar to observations in *Kcna1* ^–/–^ mice. The behavior of *Kcna1* ^–/–^ single‐mutant mice with a mixed genetic background closely resembled the previous results above for *Kcna1* ^–/–^ mice in a Tac:N:NIHS‐BC genetic background. The only difference was that the mixed genetic background unmasked sociability deficits in *Kcna1* ^–/–^ mice, evidenced by a lack of preference for the stranger chamber (two‐tailed Student's *t* test, t(16) = 0.14, *p* = .892; Figure 4a) and equal time spent sniffing the stranger mouse and the empty cup (two‐tailed Student's *t* test, t(16) = 0.36, *p* = .72; Figure 4a).

**Table 1:** Summary of behavioral phenotypes by genotype

		Tac:N:NIHS‐BC background	Mixed Tac:N:NIHS‐BC x C57BL/6J background				
		*Kcna1* ^–/–^	*Kcna1* ^–/–^	*Kcna1* ^+/–^	*Scn2a* ^+/–^	*Scn2a* ^+/–^ ; *Kcna1* ^–/–^	*Scn2a* ^+/–^ ; *Kcna1* ^+/–^
Repetitive behaviors	Marble burying			Ø			
	Nestlet shredding				Ø		Ø
	Grooming		Ø			Ø	Ø
Social interactions	Sociability	Ø			Ø	Ø	Ø
	Social novelty	Ø	Ø	Ø			Ø

Notes.

Downward red arrowheads () and upward green arrowheads () indicate statistically significant decreases or increases, respectively, relative to WT controls. Circles with lines through them (Ø) indicate no significant change relative to WT controls.

Interestingly, nonepileptic heterozygous *Kcna1* ^+/–^ mice exhibited behavioral abnormalities that were similar to *Scn2a* ^+/–^ mice, suggesting that partial *Kcna1* gene deletion may cause haploinsufficiency for autistic‐like phenotypes. With regard to repetitive behaviors, *Kcna1* ^+/–^ mice showed about 50% increases in grooming behavior (1‐way ANOVA, *F*(5,58) = 11.70, *p* < .0001; Tukey's post hoc, *q*(58) = 4.38, *p* = .0341; Figure 3c) relative to WT, which was comparable to the increases in *Scn2a* ^+/–^ mice. In addition, they also exhibited 73% reductions in nestlet shredding (1‐way ANOVA, *F*(5,58) = 9.17, *p* < .0001; Tukey's post hoc, *q*(58) = 4.91, *p* = .0121; Figure 3b) versus WT, a deficit not present in *Scn2a* ^+/–^ mice. However, marble burying in *Kcna1* ^+/–^ mice was normal, but in *Scn2a* ^+/–^ mice it was decreased. Whereas *Scn2a* ^+/–^ mice exhibited abnormal social interactions manifested as a lack of interest in social novelty, *Kcna1* ^+/–^ mice displayed a lack of sociability showing no significant preference for the stranger mouse when measured as either chamber time or sniffing. Thus, double‐mutant *Scn2a* ^+/–^ *; Kcna1* ^+/–^ mice represent a unique situation in which autistic‐like features are mutually ameliorated by the combination of two gene mutations that by themselves produce autistic‐like behavioral deficits.

## 4 DISCUSSION

This study uncovers a potential new role for the *Kcna1* gene in ASD pathophysiology. *Kcna1* deletion modified ASD‐relevant repetitive and social behaviors in mice in a gene dosage‐dependent manner. Complete gene deletion in *Kcna1* ^–/–^ mice led to drastic reductions in all repetitive behaviors tested, including decreased grooming, marble burying, and nestlet shredding. In contrast, partial gene deletion in heterozygous *Kcna1* ^+/–^ mice produced an opposite behavioral phenotype consistent with ASD, including increased grooming and decreased sociability. Taken together, these findings suggest that Kv1.1 subunits are important in pathways and neural networks underlying ASD and that *Kcna1* may be a modifier gene for ASD susceptibility. Interestingly, double‐mutant mice that are heterozygous for the *Kcna1* deletion mutation and carry the ASD‐associated *Scn2a* ^+/–^ mutation (i.e., *Scn2a* ^+/–^ *; Kcna1* ^+/–^) exhibited an ameliorated ASD phenotype due to potential reciprocal masking interactions between the two genes. Thus, Kv1.1 subunits may also represent a potential therapeutic target for ASD due to *Scn2a* mutations. These findings illustrate the complexity of genotype–phenotype relationships in ASD, demonstrating that genetic variants that increase disease risk can also exert beneficial effects depending on the genetic milieu.

The ability of *Kcna1* and *Scn2a* mutations, which both cause ASD‐like behaviors, to mutually suppress one another resulting in a double‐mutant mouse with relatively normal behavior underscores the importance of elucidating protective modifier interactions for improving genomic risk assessment and identifying new therapeutic targets. ASD has a significant and complex genetic etiology often involving interactions between multiple loci, such as the up to 1,000 genes estimated to influence ASD susceptibility (Krumm et al., 2015; Pickles et al., 1995; Risch et al., 1999; Spratt et al., 2019). Although genetic risk factors are usually thought of in the negative sense, as deleterious variants that add together to cause disease when a threshold is crossed, some genetic variants may be beneficial modifiers that reduce disease risk. Clinical studies of ASD leave room for the possible presence of these protective variants. For example, the most common genetic anomaly associated with ASD is deletion of the 16p11.2 chromosomal region; however, ~75% of carriers are asymptomatic for the disease, including carrier parents of children with ASD (Hanson et al., 2015). Mutations in the *SHANK2* gene are another prevalent molecular cause of ASD in patients; however, many individuals have been identified that carry potentially deleterious *SHANK2* mutations without any apparent psychiatric disorder, leading to the hypothesis that these healthy individuals may possess counteracting suppressor mutations (Leblond et al., 2012).

Mouse models of ASD also support the possibility of these genetic masking effects. In *Fmr1* knockout mice, deletion of the metabotropic glutamate receptor gene *mGlur5* rescues fragile X syndrome (FXS)‐related neurobehavioral phenotypes (Dölen et al., 2007). In *Kcna1* ^–/–^ mice, several second‐site mutations, including *Scn2a* ^+/–^, have been identified that ameliorate seizure severity and prolong lifespan (Glasscock et al., 2007; Holth et al., 2013; Mishra et al., 2017). Interestingly, in double‐mutant mice carrying the epilepsy‐causing *Kcna1* ^–/–^ mutation in addition to another epileptogenic mutation in the P/Q‐type calcium channel gene *Cacna1a* (*Cacna1a^tottering^*), epilepsy was mutually suppressed (Glasscock et al., 2007). Similarly, this work finds masking of ASD‐related features in mice by combining two ion channel mutations associated with ASD‐like behaviors. However, additional work will be needed to identify where exactly in the brain *Scn2a* and *Kcna1* mutations interact to affect neuronal function. Two possibilities are the neocortex and hippocampus where both Nav1.2 and Kv1.1 are prominently expressed and exhibit shared subcellular distributions (Duménieu et al., 2017). For example, in neocortex Nav1.2 and Kv1.1 are both expressed on axon initial segments and dendrites of pyramidal cells, whereas in hippocampus both are present on unmyelinated Schaffer collateral axons (Guan et al., 2006; Lorincz & Nusser, 2008; Monaghan et al., 2001; Spratt et al., 2019; Tian et al., 2014; Westenbroek et al., 1989). Given this potential expression overlap, coexisting mutations in the *Scn2a* and *Kcna1* genes would be expected to have dramatic but opposing complementary effects on action potential waveform characteristics (Duménieu et al., 2017).

Protective modifiers are important from a treatment standpoint since each one represents a potential therapeutic target. Variants in genes that support synaptic function are highly implicated in ASD. Therefore, a key focus of drug development in ASD has been identifying compounds that improve synaptic processes (Ghosh et al., 2013; Spooren et al., 2012). Even though ASD is a neurodevelopmental disorder, studies in mice show that symptoms can be reversed by treatment even in adulthood. Several mouse models of ASD and genetic syndromes that are highly associated with ASD (including Rett syndrome, FXS, and tuberous sclerosis) show improvement of behavioral deficits, synaptic plasticity, and cognitive measures when treated in adulthood (Ehninger et al., 2008; Guy et al., 2007; Mei et al., 2016; Michalon et al., 2012; Silverman et al., 2012). The genetic amelioration of *Scn2a* ^+/–^ ASD phenotypes by partial *Kcna1* deletion suggests that modulating synaptic function by targeting Kv1.1 channels may represent a new strategy for treatment of *Scn2a*‐associated ASD.

The heterozygous *Scn2a* ^+/–^ mice in our study exhibited autistic‐like deficits in repetitive behavior and social interaction that reinforce the utility of *Scn2a* ^+/–^ mice as a clinically relevant ASD model. Several groups have recently noted ASD‐like behaviors in *Scn2a* ^+/–^ mice, but with varying levels of expressivity likely due to differences in experimental conditions such as age and the types of tests employed. Léna and Mantegazza reported significant increases in repetitive behavior, including grooming and marble burying, in young (22–44 day old) but not adult *Scn2a* ^+/–^ mice (60–95 day old) (Léna & Mantegazza, 2019). They also found reductions in social communication measured as ultrasonic vocalization at all ages. The study by Léna and Mantegazza suggests that ASD‐like behaviors in *Scn2a* ^+/–^ mice tend to be age‐dependent, attenuating as the mice get older. Tatsukawa et al. examined adult *Scn2a* ^+/–^ mice (9 weeks old) and noted significant decreases in social approach behavior in the resident–intruder test when a stranger mouse was introduced to the home cage of the test mouse (Tatsukawa et al., 2019). However, adult *Scn2a* ^+/–^ mice (12 weeks old) in their study did not exhibit differences in three‐chamber tests of sociability and social novelty. Finally, Spratt et al. tested for ASD‐like behavior in adult *Scn2a* ^+/–^ mice (3–4 months old) and found no major deficits in nesting, grooming, and two‐chamber social interaction tests between the test mouse and a stranger mouse (Spratt et al., 2019). Phenotypic differences between the *Scn2a* ^+/–^ mice in our study and the others could be largely attributed to differences in age and genetic background. Our animals were tested at a young age (28–34 day) when ASD‐like behaviors in *Scn2a* ^+/–^ mice are the most robust, whereas most of the other studies were performed in adults. Furthermore, the animals in our experiments were tested on a mixed Tac:N:NIHS‐BC x C57BL/6J genetic background compared to a straight C57BL/6J background in the other studies. Despite some differences in results between studies, the pattern that emerges is that *Scn2a* ^+/–^ mice display age‐dependent deficits in repetitive and social behavior consistent with a role for the gene in ASD pathophysiology.

In addition to the repetitive and social behavioral abnormalities identified in this work, *Kcna1* mutant mice exhibit several other characteristics observed in ASD, suggesting that Kv1.1 subunits contribute to networks or pathways underlying autism. Children with autism have a higher prevalence of sleep problems including reduced sleep duration, increased night waking, increased rapid eye movement (REM) sleep latency, and increased overall sleep latency (Richdale & Schreck, 2009). Similarly, *Kcna1* ^–/–^ mice show drastic reductions in sleep duration, including more fragmented sleep, increased latency to REM sleep, a nearly complete absence of REM sleep, and increased number of wake epochs (Dhaibar et al., 2019; Roundtree et al., 2016). About 9% of autistic children exhibit brain overgrowth with enlargements in the cerebral hemispheres, cerebellum, caudate nucleus, and in some studies the amygdala and hippocampus (Sacco et al., 2015; Schumann et al., 2004). In mice, deletion or truncation of *Kcna1* causes megalencephaly due to enlargement of the hippocampus and ventral cortex beginning during the first postnatal month (Diez et al., 2003; Persson et al., 2007). In autism, one of the most consistently abnormal brain regions is the cerebellum, and one of the most common microscopic findings is a decrease in the number of Purkinje cells, the sole source of output from the cerebellar cortex (Fatemi et al., 2012). Kv1.1 channels are prominently expressed in the cerebellum, including high levels in basket cell axons which provide GABAergic inhibition to Purkinje cells (Wang et al., 1994). In mice, *Kcna1* deletion and missense mutations cause increased basket cell excitability leading to excessive inhibition of Purkinje cells, which can lead to stress‐induced motor dysfunction resembling the episodic ataxia in humans due to *KCNA1* mutations (Browne et al., 1994; Herson et al., 2003; Zhang et al., 1999). FXS, caused by mutations in the *FMR1* gene, is one of the most prevalent causes of monogenic autism representing about 2%–5% of all ASD diagnoses (Mila et al., 2018). In the *Fmr1* knockout mouse model of FXS, expression of Kv1.1 in the brain and neuronal Kv1.1‐mediated currents are significantly reduced in neonates and adults, suggesting FXS‐related Kv1.1 dysregulation that could contribute to ASD phenotypes (Zhu et al., 2018). Thus, the numerous ASD‐like neurobehavioral phenotypes in *Kcna1* mutant mice suggest that Kv1.1 deficiency may represent a new model for understanding autism pathomechanisms, especially in the context of epilepsy.

Recent clinical findings in epileptic encephalopathy patients point to *KCNA1* mutations being a potential molecular risk factor for ASD susceptibility. Epileptic encephalopathy (EE) refers to the process whereby early‐onset seizures and epileptiform activity impair brain function leading to cognitive, behavioral, and language deficits (Howell et al., 2016). ASD has an especially high prevalence in patients with epileptic encephalopathy. At least 34 of the 62 genes implicated in EE have also been associated with ASD, including sodium (e.g., *SCN1A* and *SCN2A*) and potassium channel genes (e.g., *KCNQ2* and *KCNQ3*), and several EE genes have ASD prevalence rates as high as 70%–100% (Srivastava & Sahin, 2017). Whole exome sequencing has now identified *KCNA1* mutations as the cause of EE in 5 child patients (Rogers et al., 2018; Verdura et al., 2019). In one patient with infantile EE, a de novo single‐nucleotide variant in *KCNA1* was found in the highly conserved Pro‐Val‐Pro motif of Kv1.1 that is essential for channel gating (Paulhus et al., 2020; Rogers et al., 2018). Interestingly, at age 4, this individual was also diagnosed with pervasive developmental disorder (PDD), which is an umbrella term for the group of neurodevelopmental disorders that includes ASD (i.e., autistic disorder, Asperger's disorder, and PDD‐not otherwise specified), childhood disintegrative disorder, and Rett's syndrome (McPartland & Volkmar, 2012; Rogers et al., 2018). Thus, the ability of *Kcna1* mutations to cause ASD‐like features in animal models and now a patient suggests that *KCNA1* should be considered as a new genetic risk factor for ASD phenotypes.

Sex‐specific differences in behavior and genetic interactions in our work cannot be ruled out because mice of both sexes were grouped together for analyses of each genotype to achieve greater statistical power. The drawback to this approach is that if behavioral sex differences are present, they could influence the results. Notably, however, other studies have demonstrated that *Scn2a* ^+/–^ mice do not exhibit significant sex differences in social and repetitive behaviors (including nesting and grooming) (Spratt et al., 2019). In addition, for the genotypes with larger sample sizes in our study (e.g., 5–7 per sex), major sex differences were not apparent (Table S1). Nonetheless, in future studies, it may be important to examine potential effects of sex on the observed behavioral genetic interactions between *Kcna1* and *Scn2a*.

## 5 CONCLUSION

These findings demonstrate the importance of elucidating interactions between genetic variants to improve neuropsychiatric disease risk assessment. As shown here, even mutations that independently produce ASD‐like behavioral deficits can act as protective variants in the right genetic milieu leading to mutual normalization of ASD phenotypes. This work also sheds light on the complexity of ASD–epilepsy comorbidity, showing that epilepsy‐associated mutations (such as *Kcna1* deletion) can produce epileptic or autistic phenotypes depending on gene dosage. The presence of ASD‐like behaviors in both mice and an epileptic encephalopathy patient due to *KCNA1* mutations suggests that *KCNA1* may represent a new susceptibility gene for ASD phenotypes. In addition, the ability of partial deletion of *Kcna1* to improve ASD phenotypes in *Scn2a* ^+/–^ suggests Kv1.1 subunits as a novel target for therapy in autism due to *Scn2a* channelopathy.

## CONFLICT OF INTEREST

The authors declare they have no conflict of interest.

## AUTHOR CONTRIBUTIONS

E.G. and J.I. conceptualized the study and wrote the paper. J.I., A.P., N.M.G., and K.C. performed the experiments. E.G., J.I., and A.P. analyzed the data.

## Supporting information

Table S1

## Data Availability

The data that support the findings of this study are available from the corresponding author upon reasonable request.
