# Supplementary material for: Kv1.1 deficiency alters repetitive and social behaviors in mice and rescues autistic‐like behaviors due to Scn2a haploinsufficiency
Source: Brain Behav. 2021 Jan 23;11(4):e02041. doi: 10.1002/brb3.2041 (PMC8035482; doi:10.1002/brb3.2041)
Supplement: Supplementary file 1 — Table S1 [file BRB3-11-e02041-s001.docx]

**Table S1. Comparison of sex differences for genotypes with ≥ 5 mice per sex**

| **Genotype** | **Behavioral test** | **Males** | **Females** | **t** | **df** | **P** |
| --- | --- | --- | --- | --- | --- | --- |
| ***Kcna1*^−/−^** | No. of marbles buried | 7.7 ± 3.2 (n=5) | 7.1 ± 2.6 (n=7) | 0.15 | 10 | 0.88 |
| (Tac:N:NIHS-BC) | % Nestlet shredded | 4.3 ± 2.6 (n=5) | 8.5 ± 4.6 (n=7) | 0.85 | 10 | 0.42 |
|  | Grooming time (s) | 84 ± 41 (n=5) | 38 ± 15 (n=7) | 1.20 | 10 | 0.26 |
| **Wild-type** | No. of marbles buried | 14.9 ± 1.4 (n=6) | 13.6 ± 1.3 (n=7) | 0.67 | 11 | 0.52 |
| (Tac:N:NIHS-BC) | % Nestlet shredded | 41.2 ± 12.6 (n=6) | 51.6 ± 12.2 (n=7) | 0.59 | 11 | 0.57 |
|  | Grooming time (s) | 256 ± 54 (n=6) | 184 ± 37 (n=7) | 1.13 | 11 | 0.28 |
| ***Scn2a*^+/−^** | No. of marbles buried | 6.3 ± 2.4 (n=7) | 2.6 ± 1.4 (n=5) | 1.16 | 10 | 0.27 |
| (mixed background) | % Nestlet shredded | 65.1 ± 16.8 (n=7) | 69.3 ± 19.2 (n=5) | 0.16 | 10 | 0.87 |
|  | Grooming time (s) | 352 ± 67 (n=7) | 363 ± 104 (n=5) | 0.09 | 10 | 0.92 |
|  | Sociability S1 chamber time (s) | 259 ± 25 (n=5) | 326 ± 77 (n=5) | 0.83 | 8 | 0.43 |
|  | Sociability E chamber time (s) | 195 ± 51 (n=5) | 173 ± 48 (n=5) | 0.30 | 8 | 0.77 |
|  | Sociability S1 sniffing time (s) | 163 ± 20 (n=5) | 152 ± 38 (n=5) | 0.27 | 8 | 0.79 |
|  | Sociability E sniffing time (s) | 93 ± 29 (n=5) | 32 ± 19 (n=5) | 1.74 | 8 | 0.12 |
|  | Social novelty S1 chamber time (s) | 333 ± 17 (n=5) | 290 ± 26 (n=5) | 1.38 | 8 | 0.20 |
|  | Social novelty S2 chamber time (s) | 212 ± 31 (n=5) | 177 ± 41 (n=5) | 0.68 | 8 | 0.51 |
|  | Social novelty S1 sniffing time (s) | 84 ± 25 (n=5) | 74 ± 26 (n=5) | 0.26 | 8 | 0.80 |
|  | Social novelty S2 sniffing time (s) | 89 ± 32 (n=5) | 79 ± 19 (n=5) | 0.28 | 8 | 0.79 |
| ***Scn2a*^+/−^; *Kcna1*^+/−^** | No. of marbles buried | 4.5 ± 1.4 (n=6) | 3.5 ± 1.0 (n=6) | 0.58 | 10 | 0.57 |
| (mixed background) | % Nestlet shredded | 36.3 ± 12.3 (n=6) | 59.4 ± 14.4 (n=6) | 1.22 | 10 | 0.25 |
|  | Grooming time (s) | 200 ± 36 (n=6) | 159 ± 39 (n=6) | 0.78 | 10 | 0.45 |

Values are expressed as means ± SEM, followed by the sample size in parentheses. Genetic background is indicated in parentheses after genotype. A mixed background means a combination of Tac:N:NIHS-BC and C57BL/6J as described in the Methods section. Statistical analysis performed using unpaired 2-tailed Student’s *t* test.
